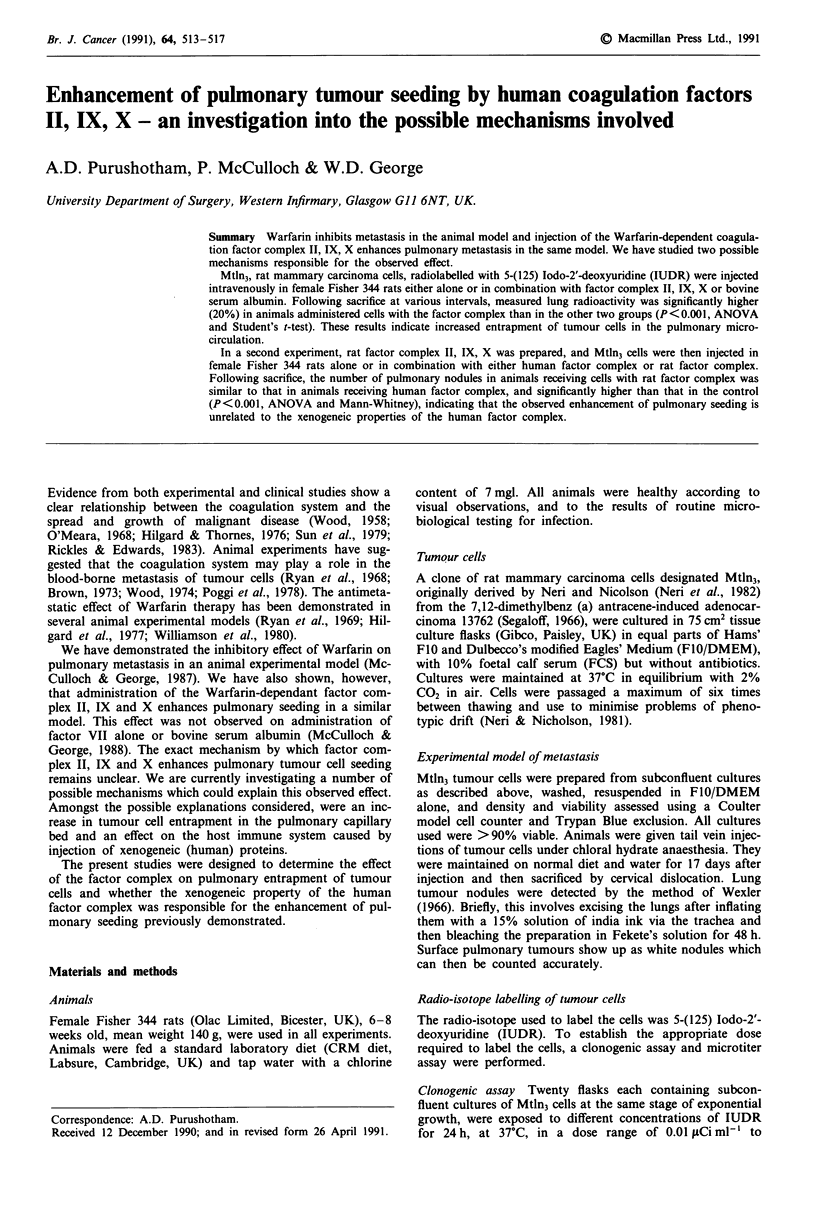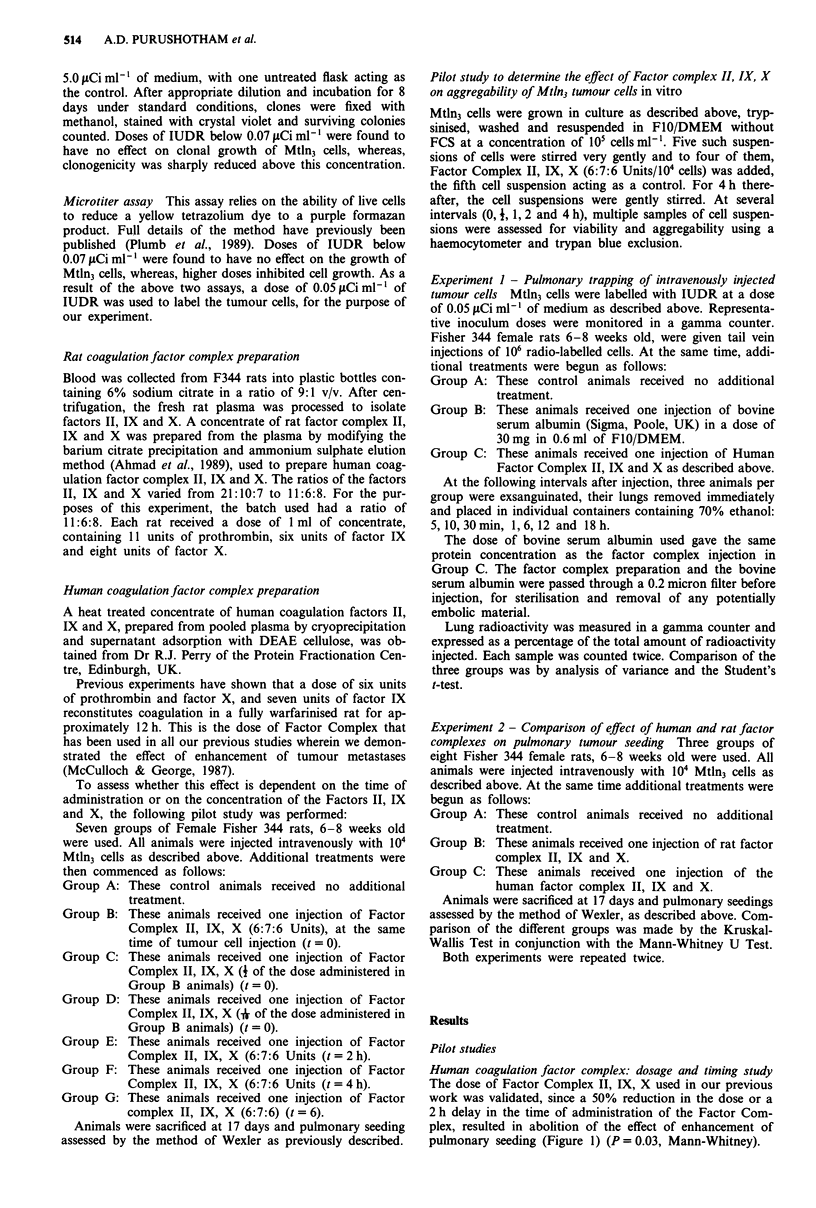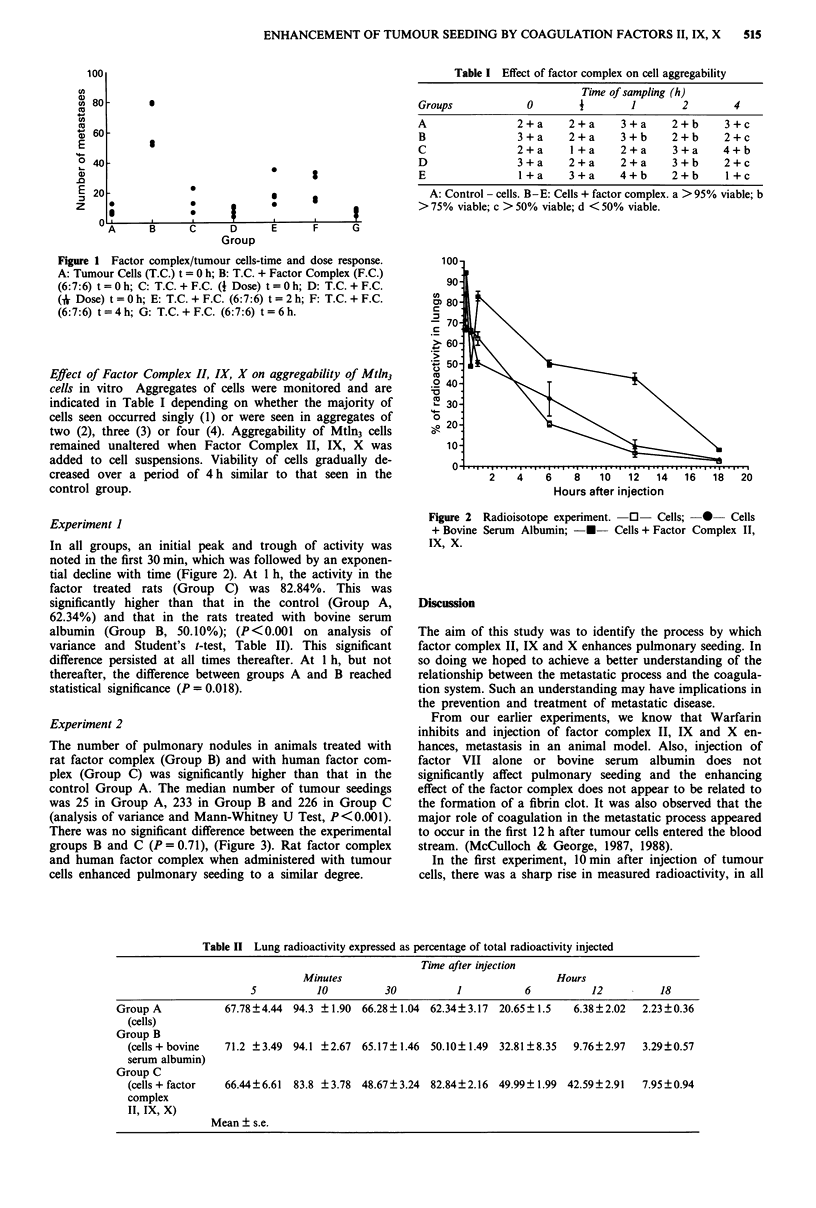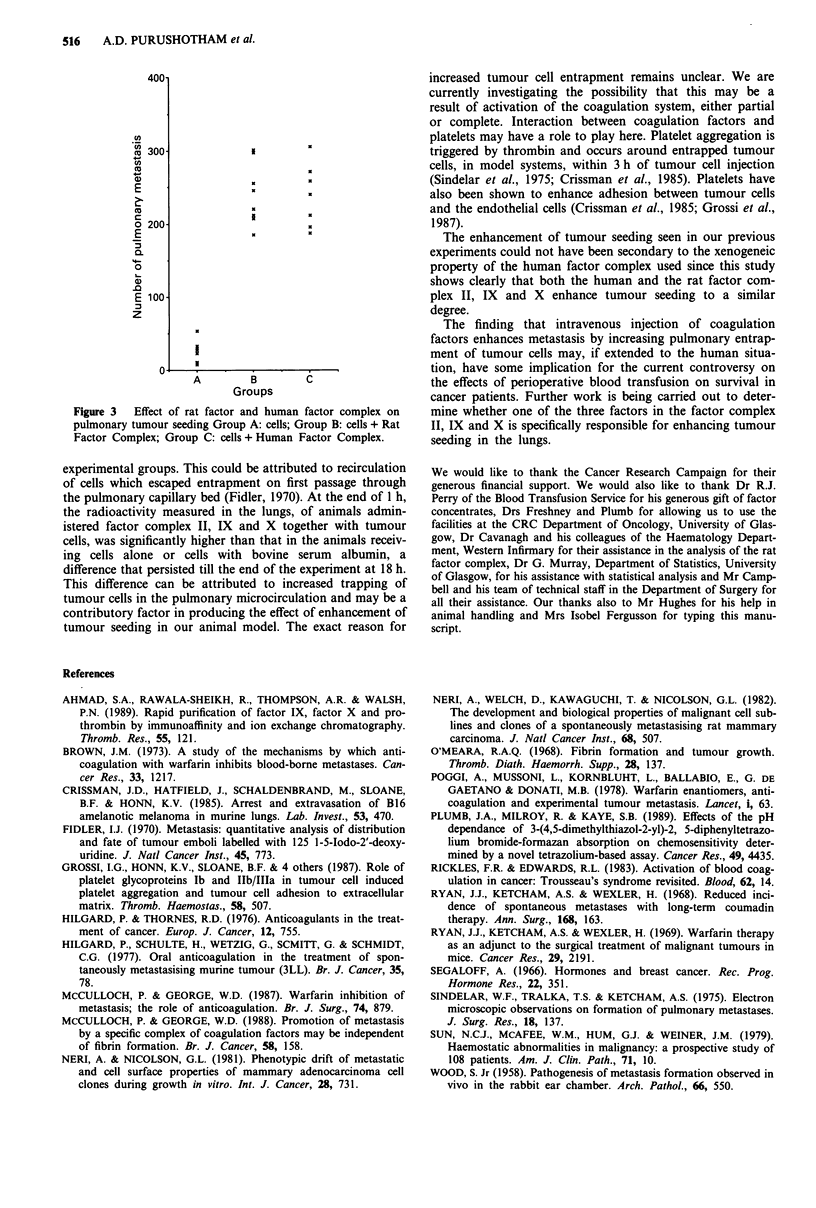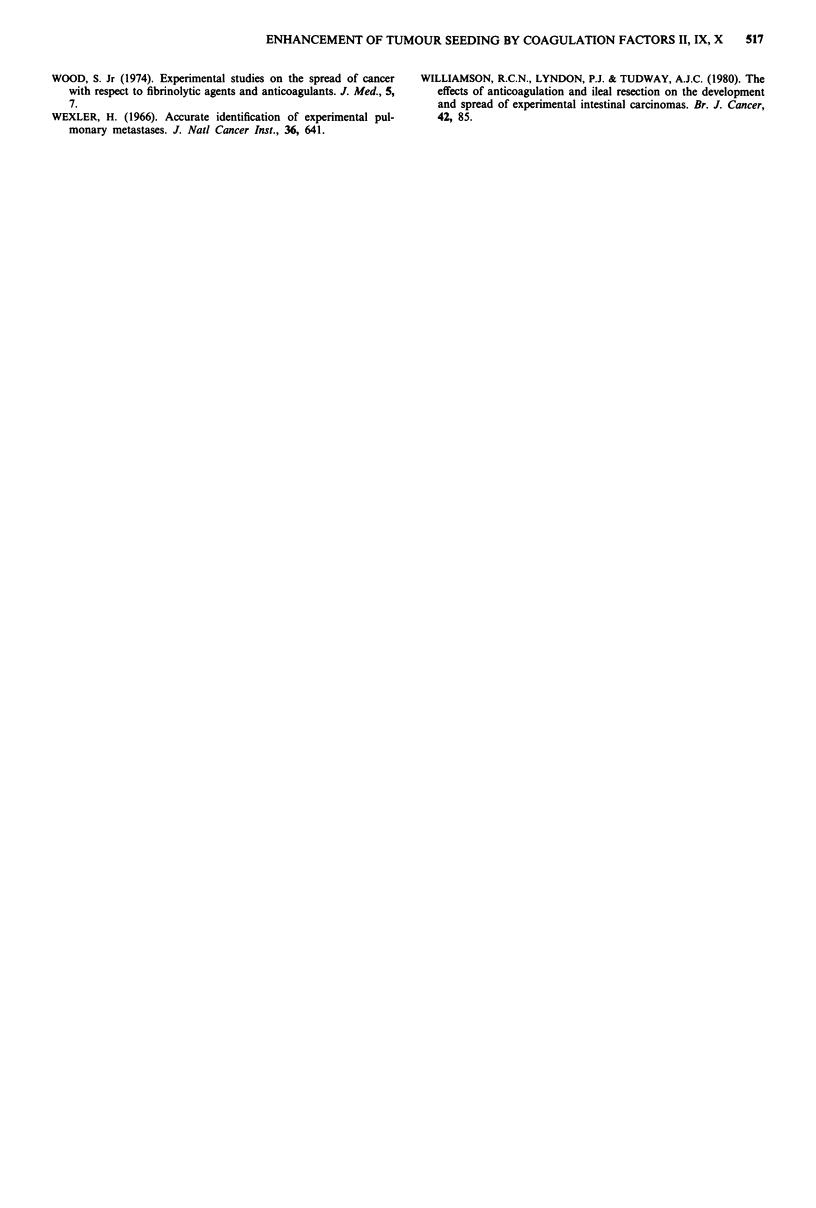# Enhancement of pulmonary tumour seeding by human coagulation factors II, IX, X--an investigation into the possible mechanisms involved.

**DOI:** 10.1038/bjc.1991.340

**Published:** 1991-09

**Authors:** A. D. Purushotham, P. McCulloch, W. D. George

**Affiliations:** University Department of Surgery, Western Infirmary, Glasgow, UK.

## Abstract

Warfarin inhibits metastasis in the animal model and injection of the Warfarin-dependent coagulation factor complex II, IX, X enhances pulmonary metastasis in the same model. We have studied two possible mechanisms responsible for the observed effect. Mtln3, rat mammary carcinoma cells, radiolabelled with 5-(125) Iodo-2'-deoxyuridine (IUDR) were injected intravenously in female Fisher 344 rats either alone or in combination with factor complex II, IX, X or bovine serum albumin. Following sacrifice at various intervals, measured lung radioactivity was significantly higher (20%) in animals administered cells with the factor complex than in the other two groups (P less than 0.001, ANOVA and Student's t-test). These results indicate increased entrapment of tumour cells in the pulmonary microcirculation. In a second experiment, rat factor complex II, IX, X was prepared, and Mtln3 cells were then injected in female Fisher 344 rats alone or in combination with either human factor complex or rat factor complex. Following sacrifice, the number of pulmonary nodules in animals receiving cells with rat factor complex was similar to that in animals receiving human factor complex, and significantly higher than that in the control (P less than 0.001, ANOVA and Mann-Whitney), indicating that the observed enhancement of pulmonary seeding is unrelated to the xenogeneic properties of the human factor complex.


Br. J. Cancer (1991), 64, 513-517 © Macmillan Press Ltd., 1991

### Enhancement of pulmonary tumour seeding by human coagulation factors II, IX, X – an investigation into the possible mechanisms involved

A.D. Purushotham, P. McCulloch & W.D. George

*University Department of Surgery, Western Infirmary, Glasgow G11 6NT, UK.*

**Summary** Warfarin inhibits metastasis in the animal model and injection of the Warfarin-dependent coagulation factor complex II, IX, X enhances pulmonary metastasis in the same model. We have studied two possible mechanisms responsible for the observed effect.

$Mtln_3$, rat mammary carcinoma cells, radiolabelled with 5-(125) Iodo-2'-deoxyuridine (IUDR) were injected intravenously in female Fisher 344 rats either alone or in combination with factor complex II, IX, X or bovine serum albumin. Following sacrifice at various intervals, measured lung radioactivity was significantly higher (20%) in animals administered cells with the factor complex than in the other two groups ($P<0.001$, ANOVA and Student's *t*-test). These results indicate increased entrapment of tumour cells in the pulmonary microcirculation.

In a second experiment, rat factor complex II, IX, X was prepared, and $Mtln_3$ cells were then injected in female Fisher 344 rats alone or in combination with either human factor complex or rat factor complex. Following sacrifice, the number of pulmonary nodules in animals receiving cells with rat factor complex was similar to that in animals receiving human factor complex, and significantly higher than that in the control ($P<0.001$, ANOVA and Mann-Whitney), indicating that the observed enhancement of pulmonary seeding is unrelated to the xenogeneic properties of the human factor complex.

Evidence from both experimental and clinical studies show a clear relationship between the coagulation system and the spread and growth of malignant disease (Wood, 1958; O'Meara, 1968; Hilgard & Thornes, 1976; Sun *et al.*, 1979; Rickles & Edwards, 1983). Animal experiments have suggested that the coagulation system may play a role in the blood-borne metastasis of tumour cells (Ryan *et al.*, 1968; Brown, 1973; Wood, 1974; Poggi *et al.*, 1978). The antimetastatic effect of Warfarin therapy has been demonstrated in several animal experimental models (Ryan *et al.*, 1969; Hilgard *et al.*, 1977; Williamson *et al.*, 1980).

We have demonstrated the inhibitory effect of Warfarin on pulmonary metastasis in an animal experimental model (McCulloch & George, 1987). We have also shown, however, that administration of the Warfarin-dependant factor complex II, IX and X enhances pulmonary seeding in a similar model. This effect was not observed on administration of factor VII alone or bovine serum albumin (McCulloch & George, 1988). The exact mechanism by which factor complex II, IX and X enhances pulmonary tumour cell seeding remains unclear. We are currently investigating a number of possible mechanisms which could explain this observed effect. Amongst the possible explanations considered, were an increase in tumour cell entrapment in the pulmonary capillary bed and an effect on the host immune system caused by injection of xenogeneic (human) proteins.

The present studies were designed to determine the effect of the factor complex on pulmonary entrapment of tumour cells and whether the xenogeneic property of the human factor complex was responsible for the enhancement of pulmonary seeding previously demonstrated.

### Materials and methods

### *Animals*

Female Fisher 344 rats (Olac Limited, Bicester, UK), 6–8 weeks old, mean weight 140 g, were used in all experiments. Animals were fed a standard laboratory diet (CRM diet, Labsure, Cambridge, UK) and tap water with a chlorine content of 7 mgl. All animals were healthy according to visual observations, and to the results of routine microbiological testing for infection.

Correspondence: A.D. Purushotham.
Received 12 December 1990; and in revised form 26 April 1991.

### *Tumour cells*

A clone of rat mammary carcinoma cells designated $Mtln_3$, originally derived by Neri and Nicolson (Neri *et al.*, 1982) from the 7,12-dimethylbenz (a) antracene-induced adenocarcinoma 13762 (Segaloff, 1966), were cultured in 75 $cm^2$ tissue culture flasks (Gibco, Paisley, UK) in equal parts of Hams' F10 and Dulbecco's modified Eagles' Medium (F10/DMEM), with 10% foetal calf serum (FCS) but without antibiotics. Cultures were maintained at 37°C in equilibrium with 2% $CO_2$ in air. Cells were passaged a maximum of six times between thawing and use to minimise problems of phenotypic drift (Neri & Nicholson, 1981).

### *Experimental model of metastasis*

$Mtln_3$ tumour cells were prepared from subconfluent cultures as described above, washed, resuspended in F10/DMEM alone, and density and viability assessed using a Coulter model cell counter and Trypan Blue exclusion. All cultures used were >90% viable. Animals were given tail vein injections of tumour cells under chloral hydrate anaesthesia. They were maintained on normal diet and water for 17 days after injection and then sacrificed by cervical dislocation. Lung tumour nodules were detected by the method of Wexler (1966). Briefly, this involves excising the lungs after inflating them with a 15% solution of india ink via the trachea and then bleaching the preparation in Fekete's solution for 48 h. Surface pulmonary tumours show up as white nodules which can then be counted accurately.

### *Radio-isotope labelling of tumour cells*

The radio-isotope used to label the cells was 5-(125) Iodo-2'-deoxyuridine (IUDR). To establish the appropriate dose required to label the cells, a clonogenic assay and microtiter assay were performed.

*Clonogenic assay* Twenty flasks each containing subconfluent cultures of $Mtln_3$ cells at the same stage of exponential growth, were exposed to different concentrations of IUDR for 24 h, at 37°C, in a dose range of 0.01 $\mu Ci\ ml^{-1}$ to

5.0 μCi ml$^{-1}$ of medium, with one untreated flask acting as the control. After appropriate dilution and incubation for 8 days under standard conditions, clones were fixed with methanol, stained with crystal violet and surviving colonies counted. Doses of IUDR below 0.07 μCi ml$^{-1}$ were found to have no effect on clonal growth of $Mtln_3$ cells, whereas, clonogenicity was sharply reduced above this concentration.

*Microtiter assay* This assay relies on the ability of live cells to reduce a yellow tetrazolium dye to a purple formazan product. Full details of the method have previously been published (Plumb *et al.*, 1989). Doses of IUDR below 0.07 μCi ml$^{-1}$ were found to have no effect on the growth of $Mtln_3$ cells, whereas, higher doses inhibited cell growth. As a result of the above two assays, a dose of 0.05 μCi ml$^{-1}$ of IUDR was used to label the tumour cells, for the purpose of our experiment.

*Rat coagulation factor complex preparation*

Blood was collected from F344 rats into plastic bottles containing 6% sodium citrate in a ratio of 9:1 v/v. After centrifugation, the fresh rat plasma was processed to isolate factors II, IX and X. A concentrate of rat factor complex II, IX and X was prepared from the plasma by modifying the barium citrate precipitation and ammonium sulphate elution method (Ahmad *et al.*, 1989), used to prepare human coagulation factor complex II, IX and X. The ratios of the factors II, IX and X varied from 21:10:7 to 11:6:8. For the purposes of this experiment, the batch used had a ratio of 11:6:8. Each rat received a dose of 1 ml of concentrate, containing 11 units of prothrombin, six units of factor IX and eight units of factor X.

*Human coagulation factor complex preparation*

A heat treated concentrate of human coagulation factors II, IX and X, prepared from pooled plasma by cryoprecipitation and supernatant adsorption with DEAE cellulose, was obtained from Dr R.J. Perry of the Protein Fractionation Centre, Edinburgh, UK.

Previous experiments have shown that a dose of six units of prothrombin and factor X, and seven units of factor IX reconstitutes coagulation in a fully warfarinised rat for approximately 12 h. This is the dose of Factor Complex that has been used in all our previous studies wherein we demonstrated the effect of enhancement of tumour metastases (McCulloch & George, 1987).

To assess whether this effect is dependent on the time of administration or on the concentration of the Factors II, IX and X, the following pilot study was performed:

Seven groups of Female Fisher 344 rats, 6–8 weeks old were used. All animals were injected intravenously with $10^4$ $Mtln_3$ cells as described above. Additional treatments were then commenced as follows:

Group A: These control animals received no additional treatment.
Group B: These animals received one injection of Factor Complex II, IX, X (6:7:6 Units), at the same time of tumour cell injection ($t = 0$).
Group C: These animals received one injection of Factor Complex II, IX, X ($\frac{1}{2}$ of the dose administered in Group B animals) ($t = 0$).
Group D: These animals received one injection of Factor Complex II, IX, X ($\frac{1}{10}$ of the dose administered in Group B animals) ($t = 0$).
Group E: These animals received one injection of Factor Complex II, IX, X (6:7:6 Units ($t = 2$ h).
Group F: These animals received one injection of Factor Complex II, IX, X (6:7:6 Units ($t = 4$ h).
Group G: These animals received one injection of Factor complex II, IX, X (6:7:6) ($t = 6$).

Animals were sacrificed at 17 days and pulmonary seeding assessed by the method of Wexler as previously described.

*Pilot study to determine the effect of Factor complex II, IX, X on aggregability of $Mtln_3$ tumour cells* in vitro

$Mtln_3$ cells were grown in culture as described above, trypsinised, washed and resuspended in F10/DMEM without FCS at a concentration of $10^5$ cells ml$^{-1}$. Five such suspensions of cells were stirred very gently and to four of them, Factor Complex II, IX, X (6:7:6 Units/$10^4$ cells) was added, the fifth cell suspension acting as a control. For 4 h thereafter, the cell suspensions were gently stirred. At several intervals (0, $\frac{1}{2}$, 1, 2 and 4 h), multiple samples of cell suspensions were assessed for viability and aggregability using a haemocytometer and trypan blue exclusion.

*Experiment 1 – Pulmonary trapping of intravenously injected tumour cells* $Mtln_3$ cells were labelled with IUDR at a dose of 0.05 μCi ml$^{-1}$ of medium as described above. Representative inoculum doses were monitored in a gamma counter. Fisher 344 female rats 6–8 weeks old, were given tail vein injections of $10^6$ radio-labelled cells. At the same time, additional treatments were begun as follows:

Group A: These control animals received no additional treatment.
Group B: These animals received one injection of bovine serum albumin (Sigma, Poole, UK) in a dose of 30 mg in 0.6 ml of F10/DMEM.
Group C: These animals received one injection of Human Factor Complex II, IX and X as described above.

At the following intervals after injection, three animals per group were exsanguinated, their lungs removed immediately and placed in individual containers containing 70% ethanol: 5, 10, 30 min, 1, 6, 12 and 18 h.

The dose of bovine serum albumin used gave the same protein concentration as the factor complex injection in Group C. The factor complex preparation and the bovine serum albumin were passed through a 0.2 micron filter before injection, for sterilisation and removal of any potentially embolic material.

Lung radioactivity was measured in a gamma counter and expressed as a percentage of the total amount of radioactivity injected. Each sample was counted twice. Comparison of the three groups was by analysis of variance and the Student's *t*-test.

*Experiment 2 – Comparison of effect of human and rat factor complexes on pulmonary tumour seeding* Three groups of eight Fisher 344 female rats, 6–8 weeks old were used. All animals were injected intravenously with $10^4$ $Mtln_3$ cells as described above. At the same time additional treatments were begun as follows:

Group A: These control animals received no additional treatment.
Group B: These animals received one injection of rat factor complex II, IX and X.
Group C: These animals received one injection of the human factor complex II, IX and X.

Animals were sacrificed at 17 days and pulmonary seedings assessed by the method of Wexler, as described above. Comparison of the different groups was made by the Kruskal-Wallis Test in conjunction with the Mann-Whitney U Test.

Both experiments were repeated twice.

### Results

*Pilot studies*

*Human coagulation factor complex: dosage and timing study* The dose of Factor Complex II, IX, X used in our previous work was validated, since a 50% reduction in the dose or a 2 h delay in the time of administration of the Factor Complex, resulted in abolition of the effect of enhancement of pulmonary seeding (Figure 1) ($P = 0.03$, Mann-Whitney).

**Figure 1** Factor complex/tumour cells-time and dose response. A: Tumour Cells (T.C.) t = 0 h; B: T.C. + Factor Complex (F.C.) (6:7:6) t = 0 h; C: T.C. + F.C. ($\frac{1}{2}$ Dose) t = 0 h; D: T.C. + F.C. ($\frac{1}{10}$ Dose) t = 0 h; E: T.C. + F.C. (6:7:6) t = 2 h; F: T.C. + F.C. (6:7:6) t = 4 h; G: T.C. + F.C. (6:7:6) t = 6 h.

*Effect of Factor Complex II, IX, X on aggregability of $Mtln_3$ cells* in vitro Aggregates of cells were monitored and are indicated in Table I depending on whether the majority of cells seen occurred singly (1) or were seen in aggregates of two (2), three (3) or four (4). Aggregability of $Mtln_3$ cells remained unaltered when Factor Complex II, IX, X was added to cell suspensions. Viability of cells gradually decreased over a period of 4 h similar to that seen in the control group.

*Experiment 1*

In all groups, an initial peak and trough of activity was noted in the first 30 min, which was followed by an exponential decline with time (Figure 2). At 1 h, the activity in the factor treated rats (Group C) was 82.84%. This was significantly higher than that in the control (Group A, 62.34%) and that in the rats treated with bovine serum albumin (Group B, 50.10%); ($P < 0.001$ on analysis of variance and Student's $t$-test, Table II). This significant difference persisted at all times thereafter. At 1 h, but not thereafter, the difference between groups A and B reached statistical significance ($P = 0.018$).

*Experiment 2*

The number of pulmonary nodules in animals treated with rat factor complex (Group B) and with human factor complex (Group C) was significantly higher than that in the control Group A. The median number of tumour seedings was 25 in Group A, 233 in Group B and 226 in Group C (analysis of variance and Mann-Whitney U Test, $P < 0.001$). There was no significant difference between the experimental groups B and C ($P = 0.71$), (Figure 3). Rat factor complex and human factor complex when administered with tumour cells enhanced pulmonary seeding to a similar degree.

**Table I** Effect of factor complex on cell aggregability

| Groups | *Time of sampling (h)* 0 | ½ | 1 | 2 | 4 |
|---|---|---|---|---|---|
| A | 2 + a | 2 + a | 3 + a | 2 + b | 3 + c |
| B | 3 + a | 2 + a | 3 + b | 2 + b | 2 + c |
| C | 2 + a | 1 + a | 2 + a | 3 + a | 4 + b |
| D | 3 + a | 2 + a | 2 + a | 3 + b | 2 + c |
| E | 1 + a | 3 + a | 4 + b | 2 + b | 1 + c |

A: Control – cells. B–E: Cells + factor complex. a >95% viable; b >75% viable; c >50% viable; d <50% viable.

**Figure 2** Radioisotope experiment. —□— Cells; —●— Cells + Bovine Serum Albumin; —■— Cells + Factor Complex II, IX, X.

**Discussion**

The aim of this study was to identify the process by which factor complex II, IX and X enhances pulmonary seeding. In so doing we hoped to achieve a better understanding of the relationship between the metastatic process and the coagulation system. Such an understanding may have implications in the prevention and treatment of metastatic disease.

From our earlier experiments, we know that Warfarin inhibits and injection of factor complex II, IX and X enhances, metastasis in an animal model. Also, injection of factor VII alone or bovine serum albumin does not significantly affect pulmonary seeding and the enhancing effect of the factor complex does not appear to be related to the formation of a fibrin clot. It was also observed that the major role of coagulation in the metastatic process appeared to occur in the first 12 h after tumour cells entered the blood stream. (McCulloch & George, 1987, 1988).

In the first experiment, 10 min after injection of tumour cells, there was a sharp rise in measured radioactivity, in all

**Table II** Lung radioactivity expressed as percentage of total radioactivity injected

| | *Time after injection* *Minutes* 5 | 10 | 30 | *Hours* 1 | 6 | 12 | 18 |
|---|---|---|---|---|---|---|---|
| Group A (cells) | 67.78±4.44 | 94.3 ±1.90 | 66.28±1.04 | 62.34±3.17 | 20.65±1.5 | 6.38±2.02 | 2.23±0.36 |
| Group B (cells + bovine serum albumin) | 71.2 ±3.49 | 94.1 ±2.67 | 65.17±1.46 | 50.10±1.49 | 32.81±8.35 | 9.76±2.97 | 3.29±0.57 |
| Group C (cells + factor complex II, IX, X) | 66.44±6.61 | 83.8 ±3.78 | 48.67±3.24 | 82.84±2.16 | 49.99±1.99 | 42.59±2.91 | 7.95±0.94 |

Mean ± s.e.

**Figure 3** Effect of rat factor and human factor complex on pulmonary tumour seeding Group A: cells; Group B: cells + Rat Factor Complex; Group C: cells + Human Factor Complex.

experimental groups. This could be attributed to recirculation of cells which escaped entrapment on first passage through the pulmonary capillary bed (Fidler, 1970). At the end of 1 h, the radioactivity measured in the lungs, of animals administered factor complex II, IX and X together with tumour cells, was significantly higher than that in the animals receiving cells alone or cells with bovine serum albumin, a difference that persisted till the end of the experiment at 18 h. This difference can be attributed to increased trapping of tumour cells in the pulmonary microcirculation and may be a contributory factor in producing the effect of enhancement of tumour seeding in our animal model. The exact reason for increased tumour cell entrapment remains unclear. We are currently investigating the possibility that this may be a result of activation of the coagulation system, either partial or complete. Interaction between coagulation factors and platelets may have a role to play here. Platelet aggregation is triggered by thrombin and occurs around entrapped tumour cells, in model systems, within 3 h of tumour cell injection (Sindelar *et al.*, 1975; Crissman *et al.*, 1985). Platelets have also been shown to enhance adhesion between tumour cells and the endothelial cells (Crissman *et al.*, 1985; Grossi *et al.*, 1987).

The enhancement of tumour seeding seen in our previous experiments could not have been secondary to the xenogeneic property of the human factor complex used since this study shows clearly that both the human and the rat factor complex II, IX and X enhance tumour seeding to a similar degree.

The finding that intravenous injection of coagulation factors enhances metastasis by increasing pulmonary entrapment of tumour cells may, if extended to the human situation, have some implication for the current controversy on the effects of perioperative blood transfusion on survival in cancer patients. Further work is being carried out to determine whether one of the three factors in the factor complex II, IX and X is specifically responsible for enhancing tumour seeding in the lungs.

We would like to thank the Cancer Research Campaign for their generous financial support. We would also like to thank Dr R.J. Perry of the Blood Transfusion Service for his generous gift of factor concentrates, Drs Freshney and Plumb for allowing us to use the facilities at the CRC Department of Oncology, University of Glasgow, Dr Cavanagh and his colleagues of the Haematology Department, Western Infirmary for their assistance in the analysis of the rat factor complex, Dr G. Murray, Department of Statistics, University of Glasgow, for his assistance with statistical analysis and Mr Campbell and his team of technical staff in the Department of Surgery for all their assistance. Our thanks also to Mr Hughes for his help in animal handling and Mrs Isobel Fergusson for typing this manuscript.

### References

AHMAD, S.A., RAWALA-SHEIKH, R., THOMPSON, A.R. & WALSH, P.N. (1989). Rapid purification of factor IX, factor X and prothrombin by immunoaffinity and ion exchange chromatography. *Thromb. Res.*, **55**, 121.

BROWN, J.M. (1973). A study of the mechanisms by which anticoagulation with warfarin inhibits blood-borne metastases. *Cancer Res.*, **33**, 1217.

CRISSMAN, J.D., HATFIELD, J., SCHALDENBRAND, M., SLOANE, B.F. & HONN, K.V. (1985). Arrest and extravasation of B16 amelanotic melanoma in murine lungs. *Lab. Invest.*, **53**, 470.

FIDLER, I.J. (1970). Metastasis: quantitative analysis of distribution and fate of tumour emboli labelled with 125 1-5-Iodo-2'-deoxyuridine. *J. Natl Cancer Inst.*, **45**, 773.

GROSSI, I.G., HONN, K.V., SLOANE, B.F. & 4 others (1987). Role of platelet glycoproteins Ib and IIb/IIIa in tumour cell induced platelet aggregation and tumour cell adhesion to extracellular matrix. *Thromb. Haemostas.*, **58**, 507.

HILGARD, P. & THORNES, R.D. (1976). Anticoagulants in the treatment of cancer. *Europ. J. Cancer*, **12**, 755.

HILGARD, P., SCHULTE, H., WETZIG, G., SCHMITT, G. & SCHMIDT, C.G. (1977). Oral anticoagulation in the treatment of spontaneously metastasising murine tumour (3LL). *Br. J. Cancer*, **35**, 78.

McCULLOCH, P. & GEORGE, W.D. (1987). Warfarin inhibition of metastasis; the role of anticoagulation. *Br. J. Surg.*, **74**, 879.

McCULLOCH, P. & GEORGE, W.D. (1988). Promotion of metastasis by a specific complex of coagulation factors may be independent of fibrin formation. *Br. J. Cancer*, **58**, 158.

NERI, A. & NICOLSON, G.L. (1981). Phenotypic drift of metastatic and cell surface properties of mammary adenocarcinoma cell clones during growth *in vitro*. *Int. J. Cancer*, **28**, 731.

NERI, A., WELCH, D., KAWAGUCHI, T. & NICOLSON, G.L. (1982). The development and biological properties of malignant cell sublines and clones of a spontaneously metastasising rat mammary carcinoma. *J. Natl Cancer Inst.*, **68**, 507.

O'MEARA, R.A.Q. (1968). Fibrin formation and tumour growth. *Thromb. Diath. Haemorrh. Supp.*, **28**, 137.

POGGI, A., MUSSONI, L., KORNBLUHT, L., BALLABIO, E., G. DE GAETANO & DONATI, M.B. (1978). Warfarin enantiomers, anticoagulation and experimental tumour metastasis. *Lancet*, **i**, 63.

PLUMB, J.A., MILROY, R. & KAYE, S.B. (1989). Effects of the pH dependance of 3-(4,5-dimethylthiazol-2-yl)-2, 5-diphenyltetrazolium bromide-formazan absorption on chemosensitivity determined by a novel tetrazolium-based assay. *Cancer Res.*, **49**, 4435.

RICKLES, F.R. & EDWARDS, R.L. (1983). Activation of blood coagulation in cancer: Trousseau's syndrome revisited. *Blood*, **62**, 14.

RYAN, J.J., KETCHAM, A.S. & WEXLER, H. (1968). Reduced incidence of spontaneous metastases with long-term coumadin therapy. *Ann. Surg.*, **168**, 163.

RYAN, J.J., KETCHAM, A.S. & WEXLER, H. (1969). Warfarin therapy as an adjunct to the surgical treatment of malignant tumours in mice. *Cancer Res.*, **29**, 2191.

SEGALOFF, A. (1966). Hormones and breast cancer. *Rec. Prog. Hormone Res.*, **22**, 351.

SINDELAR, W.F., TRALKA, T.S. & KETCHAM, A.S. (1975). Electron microscopic observations on formation of pulmonary metastases. *J. Surg. Res.*, **18**, 137.

SUN, N.C.J., MCAFEE, W.M., HUM, G.J. & WEINER, J.M. (1979). Haemostatic abnormalities in malignancy: a prospective study of 108 patients. *Am. J. Clin. Path.*, **71**, 10.

WOOD, S. Jr (1958). Pathogenesis of metastasis formation observed in vivo in the rabbit ear chamber. *Arch. Pathol.*, **66**, 550.

WOOD, S. Jr (1974). Experimental studies on the spread of cancer with respect to fibrinolytic agents and anticoagulants. *J. Med.*, **5**, 7.

WEXLER, H. (1966). Accurate identification of experimental pulmonary metastases. *J. Natl Cancer Inst.*, **36**, 641.

WILLIAMSON, R.C.N., LYNDON, P.J. & TUDWAY, A.J.C. (1980). The effects of anticoagulation and ileal resection on the development and spread of experimental intestinal carcinomas. *Br. J. Cancer*, **42**, 85.